# Attention-deficit/hyperactivity disorder (ADHD): beyond the syndrome. Implications for management and treatment

**DOI:** 10.1192/j.eurpsy.2026.10568

**Published:** 2026-07-31

**Authors:** L. López Gómez-Miguel, Á. Privado Aranda, A. Gutiérrez Fernández de Velasco, M. I. Montañés Rodríguez, A. Ladera de la Oliva, E. García Martín de la Fuente, L. M. F. Oliva, R. C. Ramos, M. D. R. D. F. Rodríguez, L. P. Ayuso Morales, M. Andrade Arango

**Affiliations:** 1 Programa de Transición y Primeros Consumos; 2 Hospital Universitario de Toledo, Toledo, Spain; 3 Universidad del Rosario, Bogotá, Colombia

## Abstract

**Introduction:**

The widespread recognition of Attention-Deficit/Hyperactivity Disorder (ADHD) has led to a simplified, predominantly symptomatic approach, often initiated by non-specialized mental health staff (e.g., educators, primary care physicians). However, recent research highlights the need for a more refined perspective, emphasizing neurobiological underpinnings—executive dysfunction, arousal dysregulation, delayed reward processing—and the differentiation between primary ADHD and secondary ADHD-like presentations stemming from complex trauma, attachment disturbances, or emotional dysregulation.

**Objectives:**

To analyze the main neurobiological hypotheses underlying ADHD (executive dysfunction, arousal regulation deficits, intolerance to reward delay), to explore the clinical overlap with trauma- and attachment-related disorders during childhood and adolescence, and to examine longitudinal outcomes and differential pharmacological responses in primary versus secondary ADHD.

**Methods:**

A narrative synthesis of recent neurobiological and clinical findings was conducted, focusing on: (1) competing neurobiological models (fronto-striatal dysfunction, aberrant arousal regulation, and reward delay intolerance); (2) symptom overlap with trauma-related and attachment disorders in children and adolescents; (3) longitudinal outcomes across developmental stages; and (4) differential response to stimulant medication in primary versus secondary ADHD.

**Results:**

Evidence supports the heterogeneity of ADHD trajectories. Neurobiologically, individuals with impaired arousal regulation or delayed reward processing show distinct profiles compared to those with inhibitory deficits, suggesting that uniform pharmacological approaches may be insufficient. Longitudinal studies show that about one-third of girls and one-fourth of boys no longer meet ADHD criteria by adolescence without intervention, highlighting the role of maturational processes. Moreover, children diagnosed with ADHD often evolve into divergent adult diagnoses (e.g., mood, anxiety, substance use disorders), underscoring the nonspecificity of early symptoms. Importantly, stimulant response differs: primary ADHD typically shows robust benefit, whereas secondary ADHD-like presentations linked to trauma or relational disturbances present attenuated or paradoxical responses.

**Image 1:**
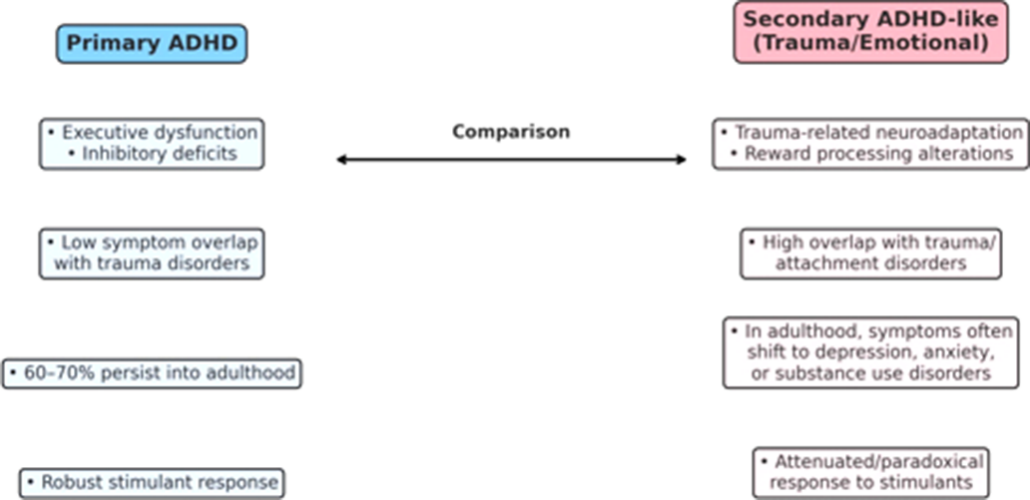
Image 1: Long description.

**Conclusions:**

Complex trauma in early development induces neuroadaptive changes in arousal and reward systems that may mimic ADHD phenotypes. Treating these cases solely with stimulants risks chronicity and progression to comorbidities such as addictions, mood, or anxiety disorders in adolescence. Therefore, a comprehensive assessment distinguishing between primary neurodevelopmental ADHD and secondary ADHD-like syndromes is crucial. Integrating trauma- and attachment-focused interventions alongside or instead of stimulants offers a more tailored, prognostically favorable approach.

**Disclosure of Interest:**

None Declared

## Long descriptions

### Image 1: Long description

A central horizontal double-headed arrow labeled Comparison separates two columns of data.

Left Column: Primary A D H D (blue header)

• Executive dysfunction and Inhibitory deficits.

• Low symptom overlap with trauma disorders.

• 60-70% persist into adulthood.

• Robust stimulant response.

Right Column: Secondary A D H D-like (Trauma/Emotional) (pink header)

• Trauma-related neuroadaptation and Reward processing alterations.

• High overlap with trauma/attachment disorders.

• In adulthood, symptoms often shift to depression, anxiety, or substance use disorders.

• Attenuated/paradoxical response to stimulants.

Navigate back to Image 1:.

